# Relationship between changes in uric acid levels and renal function during 12 months of treatment with luseogliflozin

**DOI:** 10.1007/s13340-025-00853-7

**Published:** 2025-12-03

**Authors:** Takuma Izutsu, Hiroyuki Ito, Suzuko Matsumoto, Hideyuki Inoue, Shinichi Antoku, Toshiko Mori, Sugitatsu Yoshito, Hashimoto Yo, Hashimoto Tomoko, Sugawara Takashi

**Affiliations:** 1 Department of Diabetes and Endocrinology, Iwate Prefectural Central Hospital, Iwate Prefectural Central Hospital, 1-4-1 Ueda, Moriokasi, Iwate 020-0066 Japan; 2 Department of Diabetes, Metabolism and Kidney Disease, Edogawa Hospital, Tokyo, 2-24-18 Higashikoiwa, Edogawa-ku, Tokyo-to, 133-0052 Japan

**Keywords:** Luseogliflozin, Sodium glucose cotransporter 2 inhibitors, Uric acid, Renal function, Estimated glomerular filtration rate

## Abstract

**Background:**

Sodium-glucose cotransporter-2 (SGLT2) inhibitors can effectively improve blood glucose levels in patients with type 2 diabetes, reduce the risk of cardiovascular disease and heart failure, and prevent chronic kidney disease. Although they reduce uric acid (UA) levels, few studies have investigated the relationship between UA reduction and renal function. We evaluated the degree of reduction in UA levels, various metabolic parameters, and renal function in patients with type 2 diabetes mellitus treated with luseogliflozin in order to develop a better renal protection strategy.

**Methods:**

This retrospective study analyzed 353 patients with type 2 diabetes who were newly treated with luseogliflozin. Patients were divided into two groups based on baseline UA levels, namely, the low group (< 6.0 mg/dL) and high group (≥ 6.0 mg/dL), with 74 patients in each group. Changes in metabolic parameters including glycated hemoglobin (HbA1c), UA levels, and estimated glomerular filtration rate (eGFR) were monitored over 12 months.

**Results:**

Both groups showed significant reductions in UA and HbA1c at 12 months. eGFR decreased significantly in the low group (− 3.1 ± 0.9 mL/min/1.73 m^2^, *p* = 0.002), whereas no significant change was observed in the high group (− 0.8 ± 1.3 mL/min/1.73 m^2^, *p* = 0.667). UA reduction was greater in the high group (− 1.0 ± 0.2 mg/dL vs. − 0.3 ± 0.1 mg/dL, *p* < 0.001). UA changes were significantly correlated with eGFR changes (*p* = 0.008) but not with HbA1c changes.

**Conclusion:**

Changes in eGFR after luseogliflozin administration were significantly correlated with baseline eGFR, uACR, and changes in uric acid levels (Δ uric acid). The change in uric acid levels following SGLT2 inhibitor treatment was associated with metabolic parameters such as blood pressure and albuminuria, suggesting that it may function as a surrogate marker reflecting renal metabolic processes.

## Introduction

Sodium-glucose cotransporter-2 (SGLT2) inhibitors have been reported to be beneficial not only in improving blood glucose in patients with type 2 diabetes but also in reducing the risk of cardiovascular events and heart failure [1, 2] and in preventing the progression of chronic renal failure [3, 4]. The effects are not limited to tubular SGLT2 inhibition alone but also include a variety of mechanisms that suggest beneficial effects on multiple organs [5]. The effect on uric acid levels is considered to be one such mechanism, and various types of SGLT2 inhibitors have been shown to reduce uric acid levels [6]. High serum uric acid levels are known to increase the risk of developing gouty arthritis, to have an independent adverse effect on renal function in addition to the development of renal stone disease and tubulointerstitial fibrosis, and to be an independent risk factor for developing cardiac disease [7–10]. Therefore, it is recommended that serum uric acid levels be maintained below 6.0 mg/dL in patients with a history of gout or hyperuricemia [11, 12].

Only a few studies have examined the effect of SGLT2 inhibitor therapy on uric acid levels, based on patient background and underlying uric acid levels. In addition, very few studies have examined the relationship between changes in uric acid levels and changes in glycemic control [13, 14], the effects on renal function, and various metabolic markers. We previously studied changes in renal function in patients with type 2 diabetes mellitus grouped by renal function and treated with luseogliflozin [15]. Therefore, in the present study, we investigated the relationship between changes in uric acid levels and their background factors, renal function, and other metabolic markers, including changes in glycemic control, in Japanese patients with type 2 diabetes mellitus treated with luseogliflozin for 12 months.

## Materials and methods

### Study design and patients

**Fig. 1:**
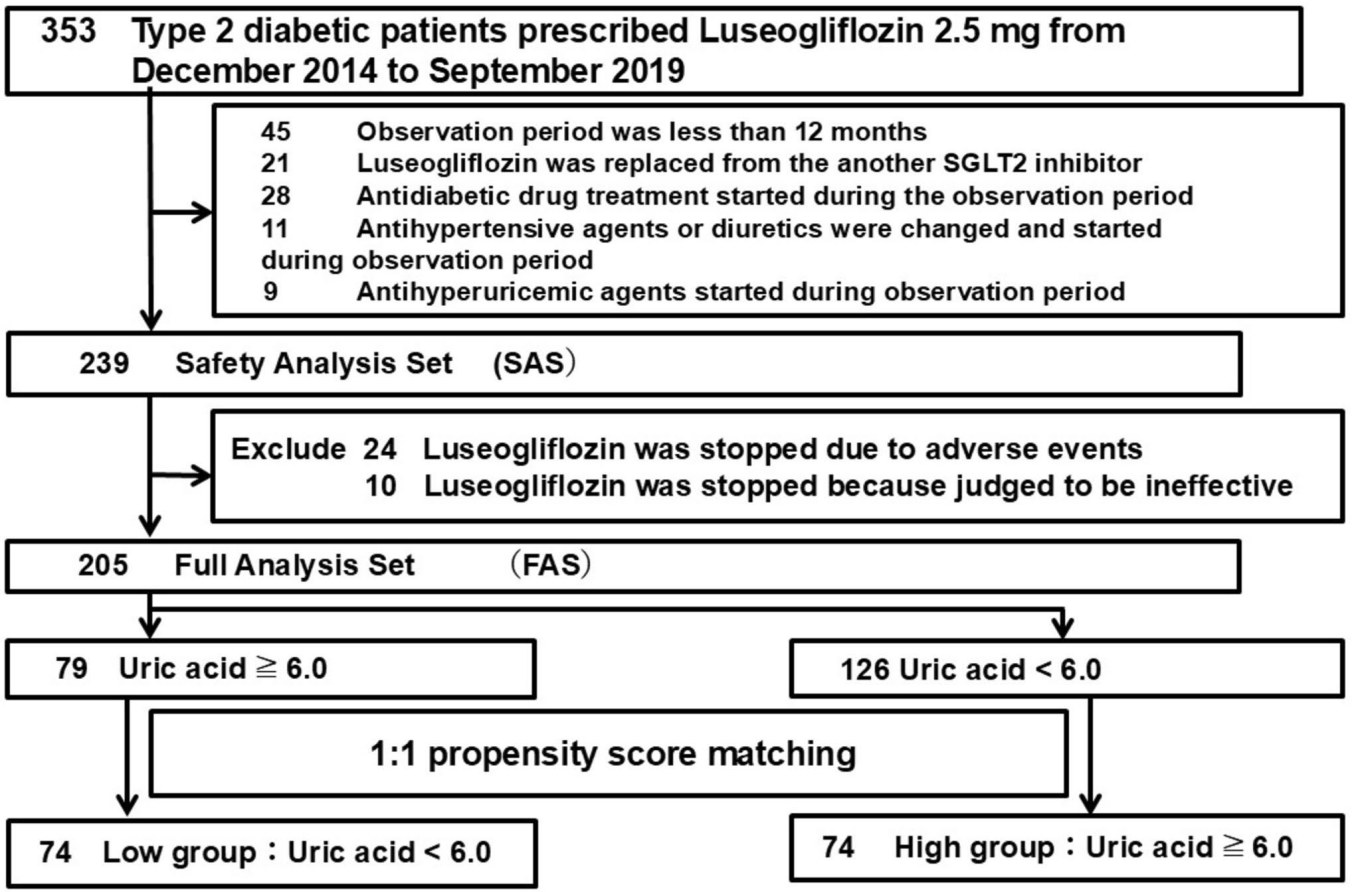
Flowchart of the patient selection process. 353 Japanese patients with type 2 diabetes mellitus receiving luseogliflozin 2.5 mg were selected; 148 patients corresponded to the exclusion criteria and 205 patients were selected for the full analysis set; FAS were assigned to two groups with baseline uric acid levels of ≥ 6.0 and < 6.0, with a 1:1 The propensity matching was performed and 74 patients were assigned to the low group and the high group, respectively

A flow chart of the patient selection process is shown in Fig. 1.

A total of 353 Japanese patients with type 2 diabetes who received 2.5 mg of luseogliflozin once daily (Lusefi® tablets; Taisho Pharmaceutical Co., Ltd., Tokyo, Japan) at our department from December 2014 to September 2019 were eligible for inclusion in this study.

The exclusion criteria were subjects in whom luseogliflozin treatment started with the replacement of another SGLT2 inhibitor (n = 21), subjects who discontinued treatment or who were transferred to other hospitals during the observation period (n = 45), subjects whose antihypertensive agents or diuretics were changed during the observation period (n = 28), subjects in whom antidiabetic drug treatment started during the observation period (n = 11), and subjects in whom antihyperuricemic agents started during the observation period (n = 9). All baseline diuretic users in this study were included in the exclusion criteria, no patients in the efficacy analysis group were using diuretics.

In total, 239 patients with type 2 diabetes (males: 71%, 59 ± 12 years old) were studied as the safety analysis set (SAS) in order to analyze the safety of luseogliflozin. After excluding subjects who discontinued luseogliflozin treatment because of any adverse events (AEs) (n = 24) or a judgement of inefficacy by physicians (n = 10), 205 subjects were investigated as the full analysis set (FAS) in order to assess the effectiveness of luseogliflozin. The SAS included 205 subjects who were divided into two groups based on their uric acid levels: a low uric acid group (n = 126) with uric acid levels < 6.0 mg/dL before luseogliflozin administration, and a high uric acid group (n = 79) with uric acid levels ≥ 6.0 mg/dL before administration. To guarantee the validity of analysis, a propensity score was applied for 1:1 matching between the low uric acid and high uric acid groups. The propensity scores were calculated using a logistic analysis including the following covariates: sex, age, duration of diabetes, current drinking, body mass index (BMI), glycated hemoglobin (HbA1c) level, estimated glomerular filtration rate (eGFR), blood pressure, urinary albumin-to-creatinine ratio (uACR), and the use of renin–angiotensin–aldosterone system (RAAS) inhibitors and antihyperuricemic agents. Based on the score of each subject, patients whose score fell within 0.03 of each other were selected at a 1:1 ratio to compare the clinical courses between the low uric acid and high uric acid groups. In total, 74 patients who were considered low uric acid and 74 who had high uric acid were investigated in this study.

### Measurements

The eGFR was calculated using the formula recommended by the Japanese Society of Nephrology. Diabetic nephropathy was defined as a uACR of ≥ 30 mg/g creatinine in a random spot urine test.

Hypertension was defined as a systolic blood pressure of ≧140 mmHg and/or a diastolic blood pressure of ≧90 mmHg. Participants currently using antihypertensive medications were also classified as being positive for hypertension. Hypercholesterolemia (elevated low-density lipoprotein (LDL) cholesterol) was defined as a serum LDL-cholesterol concentration of ≧3.62 mmol/L (140 mg/dL) or the current use of statins or ezetimibe. Hyperuricemia was defined as serum uric acid levels ≧327 μmol/L (7.0 mg/dL) or as patients using benzbromarone, allopurinol, febuxostat, or topiroxostat. A current drinker was defined as a person consuming ≧20 g ethanol equivalent/day.

Diabetic retinopathy was graded as simple, preproliferative, or proliferative retinopathy based on the results of a funduscopic examination performed by expert ophthalmologists. Diabetic peripheral neuropathy was diagnosed by the presence of two or more components among clinical symptoms (bilateral spontaneous pain, hypoesthesia, or paraesthesia of the legs), the absence of ankle tendon reflexes, and decreased vibration sensations using a C128 tuning fork.

The clinical parameters and AEs were retrospectively examined over 12 months after the initiation of luseogliflozin based on the subjects’ medical records. When clinical data including the body weight, blood pressure, HbA1c level, and serum creatinine concentration were missing, the appropriate value obtained at the previous visit was used in accordance with the last observation carried forward method.

### Statistical analyses

All data are presented as the mean ± standard deviation. The χ2 test was used for between-group comparisons of categorical variables. None of the continuous variables (age, duration of diabetes, body weight, BMI, blood pressure, uACR, HbA1c, serum lipid concentrations, aspartate transaminase [AST], alanine transaminase [ALT], γ-glutamyl transpeptidase [γ-GTP], creatinine, uric acid, and eGFR) showed a normal distribution in the Shapiro–Wilk test, so the Kruskal–Wallis test and Wilcoxon’s signed rank test were used to assess the significance of differences in the continuous variables. The χ2 test and Wilcoxon’s rank-sum test were used for between-group comparisons. Wilcoxon’s signed rank test was used to compare clinical data at the time of starting luseogliflozin and thereafter. Wilcoxon’s signed rank test was used to compare clinical data after the start of luseogliflozin. A linear regression model was used to test for factors associated with changes in clinical data after the initiation of luseogliflozin, and relationships were estimated by the least squares method. P values of < 0.05 (two-tailed) were considered to indicate statistical significance. The statistical software package JMP version 12.2.0 (SAS Institute, Cary, NC) was used to perform all analyses.

## Results

The clinical characteristics of the FAS at baseline are shown in Table 1. After matching, there were no significant differences between the two groups in age, sex, smoking history, current alcohol consumption, obesity, duration of diabetes mellitus, or percentage of cases with hypertension or hyper-LDL-C. There were also no significant differences in the HbA1c, weight, BMI, blood pressure, eGFR, uACR, AST, ALT, high-density lipoprotein cholesterol (HDL-C), and LDL-C values. Baseline uric acid levels averaged 4.9 ± 0.9 mg/dL in the low and 6.7 ± 0.5 mg/dL in the high group and were significantly higher in the high group. There were no differences between the two groups in the number of diabetes medications used, the percentage of each diabetes medication used, or the percentage of RAS inhibitors used. In the FAS, no patients in either group were using diuretics. Only allopurinol and febuxostat were used as uric acid-lowering medications, and there was no difference in their use between the two groups of 7 patients. Regarding urinary albumin, the proportion of cases with microalbuminuria was 39.8% in the low group and 39.0% in the high group, while the proportion of cases with macroalbuminuria was 5.2% in the low group and 4.5% in the high group, with no difference between the two groups. The low group had a significantly higher number of patients with coronary artery disease (*p* = 0.041), and although this difference was not significant, the low group also tended to have a higher number of patients with cerebrovascular disease (*p* = 0.097).

**Table 1:** The clinical characteristics of the full analysis set at baseline

	*n*	All subjects	Low group	High group	*p*
		n = 205	n = 74	n = 74	
Male (%)		71.4	73.4	71.9	0.841
Age (years)		59 ± 12	57.1 ± 11.5	58.5 ± 11.4	0.352
Duration of diabetes (years)		9.8	8.2 ± 6.5	9.1 ± 7.2	0.480
Smoking history (%)	n = 183	57.9	48.1	60.7	0.187
Current drinker (%)	n = 197	25.4	18.3	46.3	0.131
Body weight (kg)		72.7	78.6 ± 16.9	77.6 ± 20.7	0.588
Body mass index (kg/m^2^)		28.2	28.6 ± 5.1	28.4 ± 5.5	0.736
Obesity (%)		73.2	73.4	79.7	0.410
Systolic blood pressure (mmHg)		134.4	133.7 ± 14.5	134.1 ± 17.5	0.974
Diastolic blood pressure (mmHg)		81.0	80.6 ± 12.4	80.8 ± 12.4	0.952
Diabetic retinopathy (%)	n = 185	47.6	51.6	54.8	0.725
Diabetic nephropathy (%)	n = 199	49.2	44.4	56.2	0.199
Diabetic peripheral neuropathy (%)	n = 171	48.5	40.0	63.2	0.012
Cerebrovascular disease (%)		5.9	10.9	4.7	0.097
Coronary heart disease (%)		15.1	25.0	10.9	0.041
Peripheral arterial disease (%)		2.4	1.6	1.6	1.000
Hypertension (%)		76.1	81.3	75.0	0.396
LDL-cholesterol (mmol/L)		108.9	107.1 ± 29.3	108.8 ± 31.2	0.681
HDL-cholesterol (mmol/L)	n = 185	48.7	49.2 ± 13.2	45.3 ± 11.0	0.077
Hyper-LDL-cholesterolemia (%)		82.4	87.5	79.7	0.235
Hypo-HDL-cholesterolemia (%)	n = 185	24.9	26.6	34.4	0.349
Cholesterol lowering agents (%)		74.6	79.7	71.9	0.323
RAA inhibitor use (%)		55.6	64.1	60.9	0.720
Calcium channel blockers use (%)		43.4	42.1	53.1	0.224
Hyperuriccemia		16.1	10.9	21.9	0.106
Urate lowering agents use (%)		12.1	10.9	10.9	1.000
Febuxostat (n = 14)		6.9	6.3	6.3	1.000
Allopurinol (n = 10)		4.9	6.3	6.3	1.000
Antidaibetic agents use (%)		89.2			
Metformin		62.4	59.3	71.9	0.148
Sulfonylureas		19.0	12.5	20.3	0.231
Thiazolidinediones		12.2	12.5	9.3	0.573
α-glucosidase inhibitors		11.7	12.5	9.4	0.579
Glinides		4.9	9.4	4.7	0.311
DPP-4 inhibitors		63.4	56.3	68.8	0.157
GLP-1 receptor agonists		4.0	6.3	1.6	0.176
Insulin		24.9	21.9	25.0	0.680
Number of anti-daiabetic agents (unit)		2.0	1.9 ± 1.2	2.1 ± 1.1	0.189
uACR (mg/g Cre)		82.4 ± 180.3	60.9 ± 107.4	90.1 ± 234.8	0.712
uACR < 30 (%)		55.0	58.1	53.1	0.477
uACR 30–299 (%)		39.8	39.0	40.6	0.625
uACR > = 300 (%)		5.2	4.5	6.3	0.809
HbA1c (%)		8.3	8.0 ± 1.1	8.0 ± 1.2	0.945
HbA1c (mmol/mol)		67.1	64.2 ± 11.7	64.0 ± 12.7	0.941
AST (IU/L)		29.4	27.9 ± 16.7	31.8 ± 18.9	0.084
ALT (IU/L)		35.4	32.9 ± 21.7	39.5 ± 30.0	0.347
γGTP (IU/L)		59.1	56 ± 44.7	64.2 ± 65.1	0.630
Creatinine (μmol/L)		0.79	0.81 ± 0.28	0.82 ± 0.26	0.724
Uric acid (mg/dL)		5.3	4.9 ± 0.9	6.7 ± 0.5	< 0.001
eGFR (mL/min/1.73m^2^)		78.0	77.3 ± 19.9	75.6 ± 22.0	0.462

*RAA inhibitor* Renin Angiotensin Aldosterone system inhibitor, uACR Urinary Albumin-to-Creatinine Ratio, *eGFR* estimated glomerular filtration rate

Data are presented as mean ± standard deviation. Between-group comparisons were analyzed by the χ2 test and Wilcoxon’s rank-sum test

Table 2 shows the changes in clinical parameters of the FAS from baseline to 12 months after the initiation of luseogliflozin. Both groups showed significant reductions in uric acid levels at 12 months (low group: *p* < 0.001, high group: *p* < 0.001) compared with pre-treatment levels. Furthermore, both groups showed significant improvements in AST, ALT, γ-GTP, uACR, and HDL-C, in addition to decreases in HbA1c, weight, and BMI. However, with regard to blood pressure, only the high group showed a significant decrease in both systolic and diastolic blood pressure, while the low group showed no significant change. In terms of eGFR, propensity score matching showed that baseline eGFR before luseogliflozin was 77.3 mL/min/1.73m^2^ in the low group and 75.6 mL/min/1.73m^2^ in the high group, with no difference between the two groups (*p* = 0.462). Despite this, the 12-month change in eGFR (Δ eGFR) in the high group was − 0.8 ± 1.3 mL/min/1.73m^2^ (*p* = 0.667 vs. baseline eGFR), showing no significant change over 12 months, while the Δ eGFR from baseline in the low group was − 3.1 ± 0.9 mL/min/1 0.73m^2^ (*p* < 0.001 vs. baseline eGFR), a significantly greater decrease. However, as shown in Table 3, no significant differences were observed between the two groups in the degree of change from baseline for eGFR (*p* = 0.667). Similarly, changes in HbA1c, body weight/BMI, blood pressure, and uACR did not differ significantly between the groups. The change in uric acid levels (Δ uric acid) was the only parameter that showed a significant difference between the groups, with a greater reduction observed in the high group compared to the low group (low group: − 0.3 ± 0.1 mg/dL; high group: − 1.0 ± 0.2 mg/dL; *p* < 0.001).

**Table 2:** Changes in clinical parameters of each group

	Low group			High group			*P* (High group 12 month vs Low group 12 month)
	Baseline	12 month	*p*	Baseline	12 month	*p*	
Uric acid (mg/dL)	4.9 ± 0.9	4.6 ± 1.1	< 0.001	6.7 ± 0.5	5.6 ± 1.0	< 0.001	< 0.001
eGFR (mL/min/1.73m^2^)	77.3 ± 19.9	73.9 ± 18.5	< *0.001*	75.6 ± 22.0	74.9 ± 21.6	0.667	0.858
HbA1c (%)	8.0 ± 1.1	7.4 ± 0.9	< 0.001	8.0 ± 1.2	7.5 ± 1.0	< 0.001	0.531
uACR (mg/g Cre)	60.9 ± 107.4	53.5 ± 112.9	0.001	90.1 ± 234.8	62.3 ± 192.7	< 0.001	0.212
Systolic blood pressure (mmHg)	133.7 ± 14.5	128.8 ± 14.3	0.079	134.1 ± 17.5	129.5 ± 13.8	0.041	0.873
Diastolic blood pressure (mmHg)	80.6 ± 12.4	78.2 ± 12.1	0.080	80.8 ± 12.4	77.8 ± 11.0	0.034	0.995
Body weight (kg)	78.6 ± 16.9	76.1 ± 16.9	< 0.001	77.6 ± 20.7	75.5 ± 19.0	< 0.001	0.560
Body mass index (kg/m^2^)	28.6 ± 5.1	27.7 ± 4.9	< 0.001	28.4 ± 5.5	27.7 ± 5.2	< 0.001	0.824
LDL-cholesterol (mmol/L)	107.1 ± 29.3	98.4 ± 26.5	0.064	108.8 ± 31.2	106.5 ± 31.4	0.733	0.161
HDL-cholesterol (mmol/L)	49.2 ± 13.2	53.8 ± 16.2	< 0.001	45.3 ± 11.0	51.2 ± 14.5	< 0.001	0.316
AST (IU/L)	27.9 ± 16.7	23.4 ± 10.3	0.010	31.8 ± 18.9	28.1 ± 18.7	0.018	0.189
ALT (IU/L)	32.9 ± 21.7	26.0 ± 15.6	< 0.001	39.5 ± 30.0	30.2 ± 29.2	< 0.001	0.997
γGTP (IU/L)	56 ± 44.7	44.5 ± 34.8	< 0.001	64.2 ± 65.1	46.2 ± 41.9	< 0.001	0.839

*RAA inhibitor* Renin–Angiotensin–Aldosterone system inhibitor, *uACR* Urinary Albumin-to-Creatinine Ratio

*eGFR* estimated glomerular filtration rate

Data are mean ± standard deviation. Between-group comparisons were analyzed by the χ2 test and Wilcoxon’s rank-sum test

**Table 3:** Comparison the change of degree in clinical parameters of each group

	Low group	High group	*p*
	n = 74	n = 74	
Δ uric acid (mg/dL)	− 0.3 ± 0.1	− 1.0 ± 0.2	< 0.001
Δ eGFR (mL/min/1.73m^2^)	− 3.1 ± 0.9	− 0.8 ± 1.3	0.095
Δ HbA1c (%)	− 0.7 ± 0.1	− 0.5 ± 0.1	0.279
Δ uACR (mg/g Cre)	− 3.8 ± 3.3	− 22.4 ± 12.7	0.233
Δ Systolic blood pressure (mmHg)	− 4.4 ± 2.0	− 4.6 ± 1.9	0.714
Δ Diastolic blood pressure (mmHg)	− 2.4 ± 1.3	− 3.0 ± 1.3	0.842
Δ Body weight (kg)	− 2.4 ± 0.4	− 2.0 ± 0.5	0.601
Δ Body mass index (kg/m^2^)	− 0.9 ± 0.2	− 0.7 ± 0.2	0.622

*uACR* Urinary Albumin-to-Creatinine Ratio, *eGFR* estimated glomerular filtration rate

Δ represents the change in parameters from the baseline to 12 months of administration

Data are presented as mean ± standard deviation

**Fig. 2:**
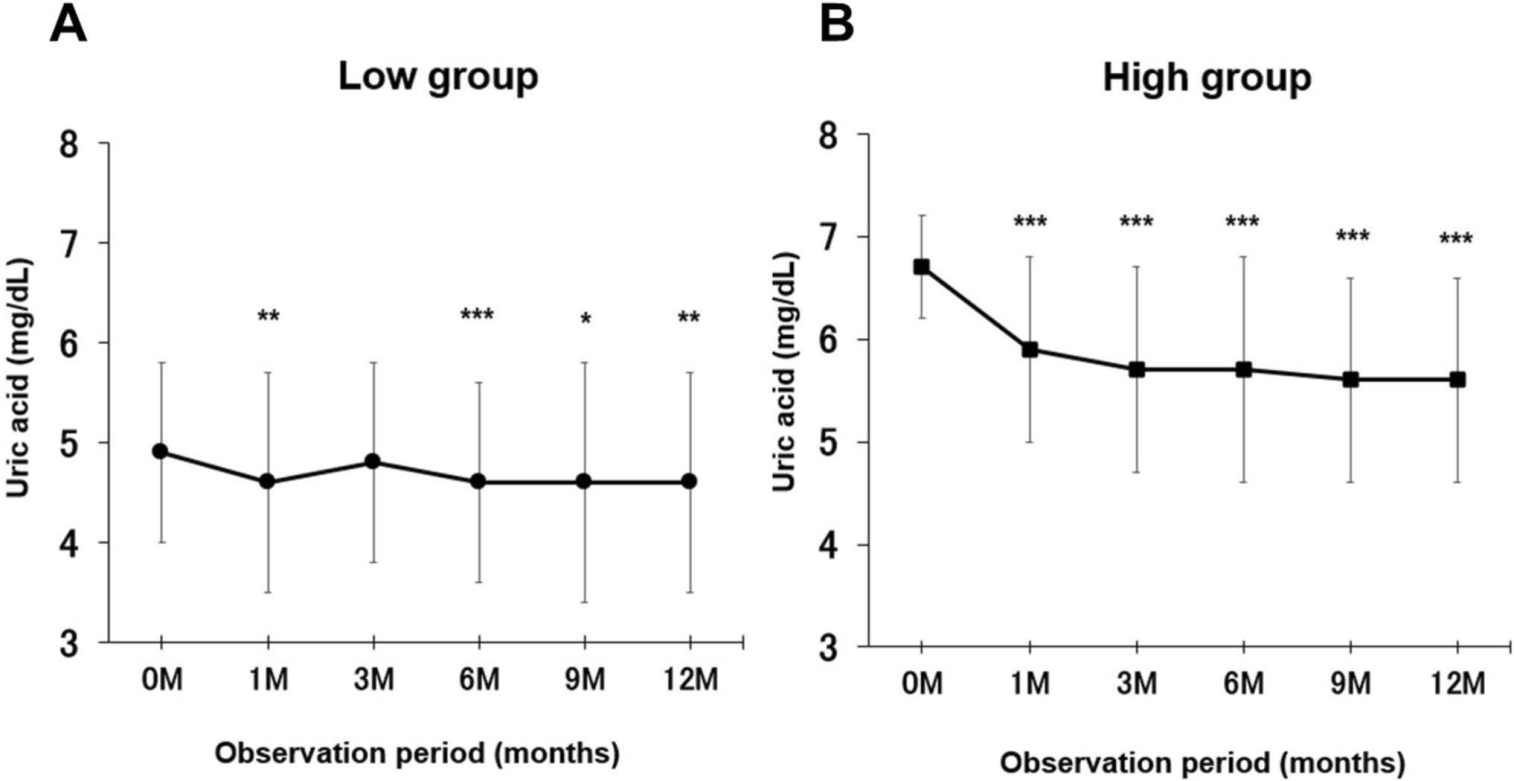
In the two subgroups (A: low group, B: high group), serum uric acid concentrations were measured at baseline and at multiple time points during the 12-month period. Both the low and high group showed significant reductions in uric acid levels starting at 1 month and persisting at 12 months. Data are presented as mean ± SD; error bars indicate SD. *P < 0.05, **P < 0.01, ***P < 0.001 vs. baseline

Figure 2 shows the trend of uric acid levels and eGFR during 12 months in both groups. In both groups, uric acid levels decreased significantly after 1 month of luseogliflozin administration and remained so for 12 months.

**Fig. 3:**
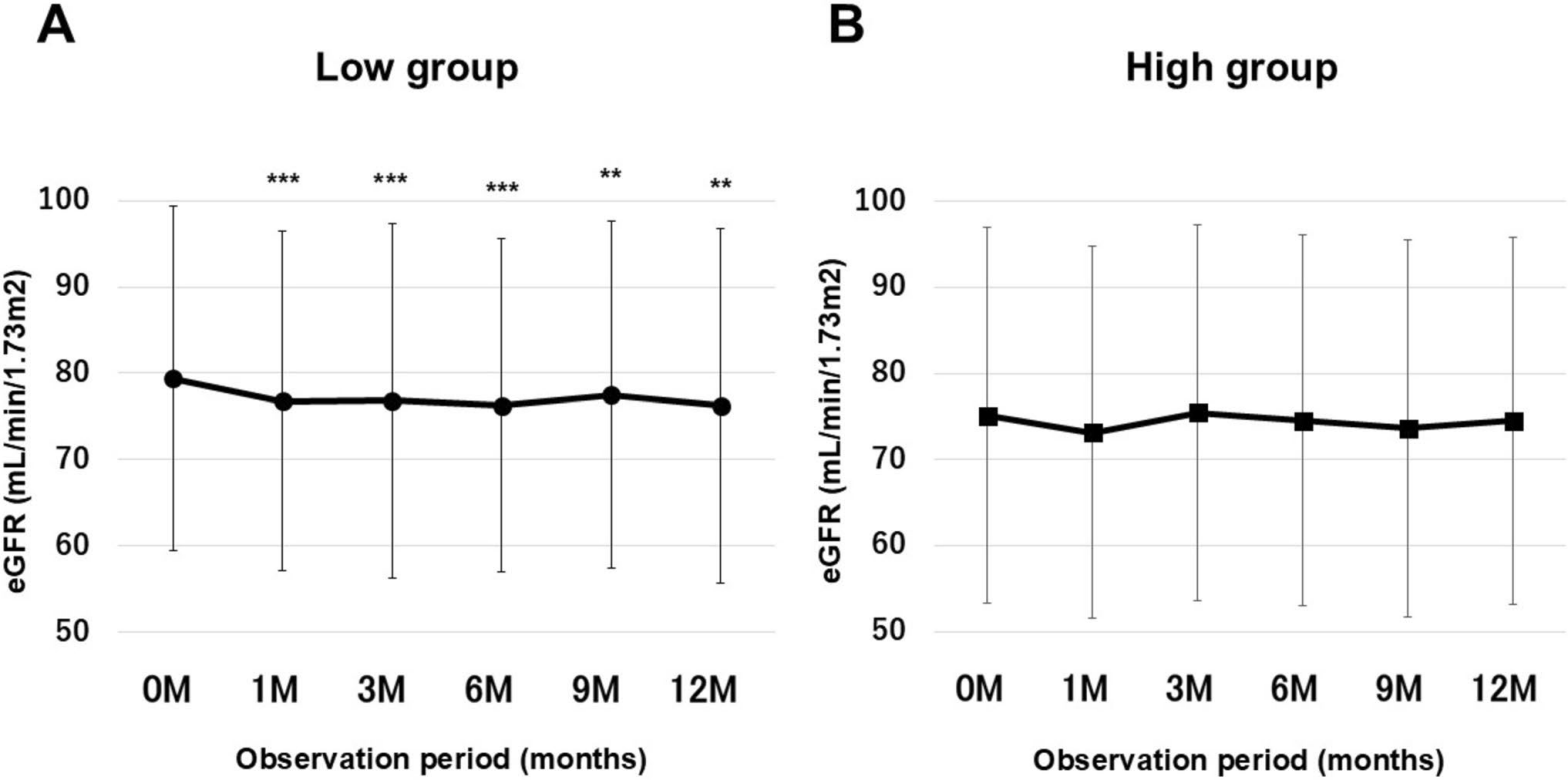
In the two subgroups (A: low group, B: high group), eGFR were measured at baseline and at multiple time points during the 12-month period. The high group showed no significant change in eGFR throughout the 12 months, while the low group showed a significant decrease in eGFR beginning at 1 month. Data are presented as mean ± SD; error bars indicate SD. *P < 0.05, **P < 0.01, ***P < 0.001 vs. baseline

Figure 3 shows the trend of eGFR. The high group showed no significant change in eGFR during the 12-month period, while the low group showed a significant decrease in eGFR from the first month of treatment and maintained the decreased level throughout the 12-month period.

Table 4 shows the results of a multivariate analysis of the FAS regarding the relationship with the baseline clinical parameters, using Δ eGFR as the objective variable. Multiple regression analysis was performed on the following explanatory variables: sex, age, smoking history, current alcohol consumption, BMI, blood pressure, baseline HbA1c, Δ HbA1c, baseline eGFR, Δ eGFR, baseline uric acid level, uACR, Δ uACR, and the use of antihyperuricemic agents. Δ eGFR was significantly affected by baseline eGFR, uACR, Δuric acid, current drinker as independent variables.

**Table 4:** Relationship between the changes in the Δ eGFR and the clinical parameters at baseline in the full analysis set

	Changes in the eGFR Multiple regression	
	Regression coefficient	*p*
Baseline eGFR (mL/min/1.73m^2^)	ー0.133	0.003
uACR (mg/g Cre)	ー0.122	0.006
Δ uric acid (mg/dL)	ー2.099	0.020
Current drinker (%)	ー2.470	0.032
Smoking history (%)	ー1.478	0.153
Baseline HbA1c (%)	ー1.025	0.172
Diastolic blood pressure (mmHg)	ー0.139	0.442
Urate lowering agents use (%)	0.011	0.466
RAA inhibitor use (%)	0.243	0.691
Systolic blood pressure (mmHg)	ー0.171	0.790
Body mass index (kg/m^2^)	ー0.034	0.845
Age (years)	ー0.156	0.924
Male (%)	ー0.195	0.924
Baseline Uric acid (μmol/L)	0.009	0.991

*RAA inhibitor* Renin–Angiotensin–Aldosterone system inhibitor, *uACR* Urinary Albumin-to-Creatinine Ratio, *eGFR* estimated glomerular filtration rate

Δ represents the change in parameters from the baseline to 12 months of administration

Table 5 is used as in Table 4, the results of a multivariate analysis of the FAS regarding the relationship with the baseline clinical parameters, using Δ uric acid as the objective variable. Δ Uric acid was significantly affected by pre-administration uric acid levels, Δ eGFR, and sex as independent variables. Δ eGFR and Δuric acid showed no correlation with the degree of change in HbA1c (Δ HbA1c). Similarly, it did not correlate with the degree of change in body weight.

**Table 5:** Relationship between the changes in the Δ uric acid and the clinical parameters at baseline in the full analysis set

	Changes in the uric acid Multiple regression	
	Regression coefficient	*p*
Male (%)	0.434	< 0.001
Baseline Uric acid (mg/dL)	− 0.441	< 0.001
ΔeGFR (mL/min/1.73m2)	− 0.036	0.012
Baseline HbA1c (%)	0.065	0.091
Smoking history (%)	0.0489	0.100
Baseline eGFR (mL/min/1.73m^2^)	− 0.012	0.137
Age (years)	− 0.019	0.361
Diastolic blood pressure (mmHg)	< 0.001	0.398
RAA inhibitor use (%)	0.134	0.429
Systolic blood pressure (mmHg)	< 0.001	0.431
Urate lowering agents use (%)	− 0.189	0.466
Current drinker (%)	0.055	0.788
uACR (mg/g Cre)	< 0.01	0.814
Body mass index (kg/m^2^)	< 0.001	0.979

*RAA inhibitor* Renin–Angiotensin–Aldosterone system inhibitor, *uACR* Urinary Albumin-to-Creatinine Ratio, *eGFR* estimated glomerular filtration rate

Δ represents the change in parameters from the baseline to 12 months of administration

**Fig. 4:**
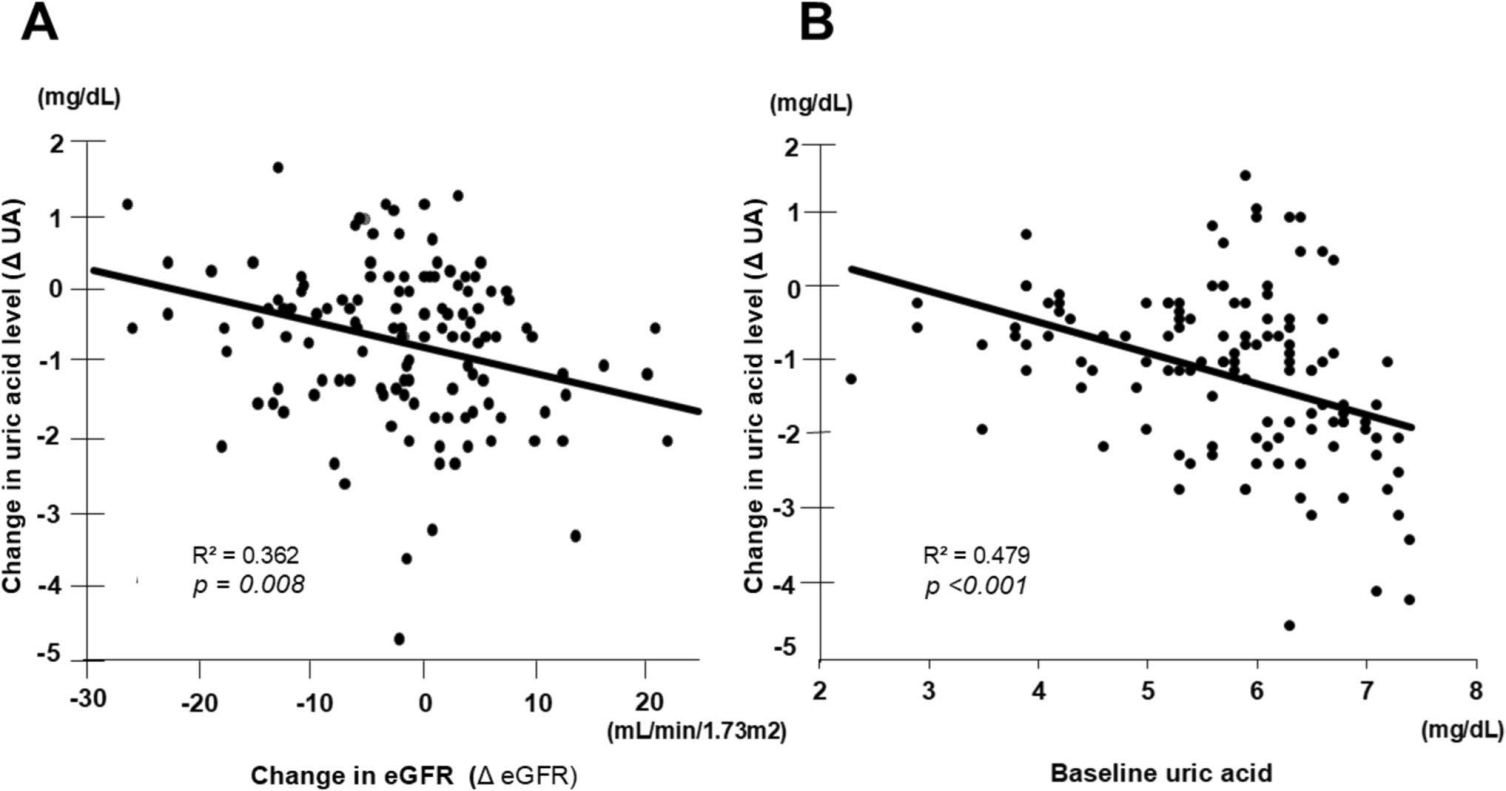
**A** Relationship between the degree of change in uric acid level (Δ uric acid) and the degree of change in eGFR (Δ eGFR). **B** Relationship between the degree of change in uric acid level and baseline uric acid level before luseogliflozin administration. (The Δ uric acid and Δ eGFR presented in **A** showed a significant correlation. Similarly, **B** Δ uric acid and baseline uric acid levels showed a significant correlation. R2 presented shows the correlation coefficient

In Fig. 4, panel A shows the relationship between Δ eGFR and Δ uric acid. The regression coefficient was − 1.08 (95% CI − 1.32 to − 0.84; *p* = 0.008; R^2^ = 0.362), indicating that greater increases in Δ uric acid were associated with smaller Δ eGFR. Panel B shows the correlation between Δ uric acid and baseline uric acid levels before luseogliflozin administration. The regression coefficient was − 0.56 (95% CI − 0.77 to − 0.37; *p* < 0.001; R^2^ = 0.479), indicating that higher baseline uric acid levels were associated with greater Δ uric acid.

We also conducted analyses excluding cases who were using antihyperuricemic agents from both the low and high uric acid groups.

In these cases, the baseline serum uric acid levels were 4.9 ± 1.2 mg/dL in the low group and 6.9 ± 0.8 mg/dL in the high group, with significantly higher levels observed in the high group (*p* < 0.001). Both groups showed significant reductions in uric acid levels at 12 months compared with their respective baseline values (low group: *p* < 0.001, high group: *p* < 0.001).

Regarding eGFR, baseline values were 76.5 ± 17.4 mL/min/1.73 m^2^ in the low group and 75.1 ± 19.8 mL/min/1.73 m^2^ in the high group, with no significant difference between the two. At 12 months, however, only the low group showed a significant change in eGFR (low group: *p* < 0.001, high group: *p* = 0.730).

In the comparison of changes in clinical parameters between the two groups shown in Table 3, the Δ uric acid was − 0.4 ± 0.2 mg/dL in the low group and − 1.0 ± 0.4 mg/dL in the high group, with a significantly greater reduction observed in the high group (p < 0.001). As for Δ eGFR, it was − 2.9 ± 0.9 mL/min/1.73 m^2^ in the low group and − 0.9 ± 1.4 mL/min/1.73 m^2^ in the high group, with no statistically significant difference between the groups (*p* = 0.101). Multivariate analysis, as shown in Table 4, revealed that Δ eGFR was significantly associated with baseline eGFR, baseline uACR, Δ uric acid, and alcohol consumption status as explanatory variables.

Furthermore, when Δ uric acid was used as the dependent variable in the multivariate analysis, as in Table 5, it was significantly affected by baseline uric acid levels, Δ eGFR, and sex as independent variables.

### Safety

In the SAS, luseogliflozin was discontinued in 24 patients (14%) because of the development of AEs. Within 100 days of starting luseogliflozin, AEs were observed that could be related to luseogliflozin administration, including urogenital infection, increased urine output, decreased fluid volume, skin rash, and gastrointestinal symptoms. In addition, luseogliflozin was stopped in 10 patients (4%) because it was judged to be ineffective at the discretion of the attending physician, but the frequency of discontinuation did not differ between the high and low groups.

## Discussion

This is the first report of a study that stratifies patients by uric acid levels, matches the patient background with metabolic parameters related to type 2 diabetes, and observes changes in metabolic factors, including changes in uric acid levels, caused by luseogliflozin, making this a noteworthy point of the study.

In the present study, changes in uric acid levels based on baseline uric acid levels and changes in eGFR in response to the changes in uric acid levels in type 2 diabetes patients treated with the SGLT2 inhibitor luseogliflozin were observed, matched to patient background. The results showed that there was a decrease in uric acid levels irrespective of the degree of improvement in glycemic control.

Examining the results of this study, both the low and high baseline uric acid groups showed significant improvement in uric acid levels with luseogliflozin, and the reduction in uric acid levels was maintained for 12 months in both groups. The degree of change in uric acid level was significantly greater in the high group, but when serum uric acid levels were compared between the two groups after 12 months, the high group's uric acid levels remained significantly higher (low group: 4.6 ± 1.1 mg/dL, high group: 5.6 ± 1.0 mg/dL, *p* < 0.001). One of the reasons for the large difference in the degree of change in uric acid levels between the two groups was that in some cases, uric acid levels even increased in the low uric acid group, likely due to regulation through URAT1 and GLUT9, which maintain serum uric acid within the range of approximately 4–6 mg/dL [16–18].

However, the decline in eGFR in the high group was − 0.8 mL/min/1.73 m^2^/year, whereas the low group showed a greater decline of − 3.1 ± 0.9 mL/min/1.73 m^2^/year, exceeding the expected annual decline from baseline eGFR (approximately − 0.7 to − 1.5 mL/min/1.73 m^2^) [19–22]. As shown in Table 1, the low group had significantly more cases with coronary artery disease (*p* = 0.041) and a tendency toward more cerebrovascular disease (*p* = 0.097), suggesting more advanced atherosclerosis. In addition, at 12 months, a ≥ 10% decline in eGFR occurred more frequently in the low group than in the high group (32% vs. 18%), and vascular comorbidities such as coronary artery disease, heart failure, and peripheral vascular disease were also more common among those with a ≥ 15% decline. Compared with previous large trials, the prevalence of albuminuria at baseline was slightly higher in our cohort, reaching approximately 45%. As shown in Tables 2 and 3, blood pressure improvement was observed only in the high group, and the degree of uACR reduction tended to be greater in this group, although not statistically significant. These factors may partly explain the greater decline in eGFR observed in the low uric acid group. Nevertheless, causality remains unproven in this study. Furthermore, as shown in Table 3, there was no significant difference in the degree of change in eGFR over 12 months between the two groups, indicating that these results should be interpreted with caution.

Following this, we also consider the correlation between Δ eGFR and Δ uric acid. In the high group, we compared cases where uric acid levels improved to below 6.0 mg/dL, the treatment target for hyperuricemia, at 12 months, with cases where they did not improve. Cases where uric acid levels improved to 6.0 mg/dL had significantly lower baseline uACR, weight were significantly lower, and baseline AST, ALT, and γ-GTP values were significantly higher. Additionally, in this group, AST, ALT, γ-GTP, HDL-C, and blood pressure were significantly improved at 12 months after luseogliflozin administration compared to baseline. As shown in Table 4, Δ eGFR also showed a significant correlation with drinking history. Particularly in the high group, where reductions in alcohol consumption and improvements in lifestyle were more pronounced, the improvement in blood pressure may have contributed to improvements in metabolic parameters including uric acid and eGFR, as it shares common effects on uric acid levels and renal function.

Improvements in liver dysfunction may contribute to improved insulin resistance and uric acid levels [23–28], in addition, improvements in the renin–angiotensin–aldosterone system (RAAS) may lead to corrected glomerular capillary pressure [29–32], thereby demonstrating renal protective effects. Therefore, the fact that many cases with improved liver enzyme levels also showed improved uric acid levels may be one reason why a correlation between changes in eGFR and uric acid levels was demonstrated. Based on these results, changes in uric acid levels after SGLT2 inhibitor administration may also serve as alternative indicators of glucose metabolism parameters and renal processes.

However, to prove this, it may be necessary to examine the long-term gradient of eGFR in a larger number of patients. Although the median eGFR in both groups in this study was in the seventies range, it has recently been reported that SGLT2 inhibitor therapy reduces uric acid levels in Grade 3 and Grade 4 CKD patients [33, 34]. Future long-term observation of Δ uric acid and Δ eGFR in each eGFR group will provide more detailed information on the relationship and mechanism between Δ uric acid and Δ eGFR, and the mechanisms in each eGFR group should be examined.

## Limitation

### Several limitations associated with the present study warrant mention

First, this study found a consistent association between changes in uric acid (UA) and changes in eGFR; however, this study is a retrospective observational study and does not prove causality. In other words, a decrease in UA does not necessarily directly lead to the maintenance of eGFR, and as noted in the discussion, it is also important to consider the possibility that UA may serve as a surrogate marker for other renal protective or metabolic factors. In fact, large-scale randomized controlled trials (RCTs) evaluating the efficacy of uric acid-lowering therapy have reported that lowering uric acid does not necessarily lead to the suppression of renal function decline [35]. Therefore, the correlation between Δ UA and Δ eGFR observed in this study may be influenced by other factors such as alcohol consumption, liver function, and blood pressure, and caution is required when interpreting it causally.

Second, as discussed in the discussion section, the rate of eGFR decline in the low group in this study may be greater than that in the general patients with type 2 diabetes cases. Therefore, it is necessary to consider the possibility that the relationship between Δ eGFR and Δ uric acid observed in this study may not hold true when examined using the eGFR slope observed in the general patients with type 2 diabetes cases.

Additionally, this study was conducted over a 12-month period. As mentioned earlier, it has been shown that some cases, such as rapid decliners, may experience a rapid decline in eGFR when SGLT2 inhibitors are administered. However, as demonstrated in large-scale clinical trials, when observing a period of approximately three years, there are also cases in which the eGFR slope does show a slow decline in cases where RAS inhibitors can be continued or where blood pressure control is good [36]. Therefore, further long-term observation is necessary in this study group as well, as new findings may be obtained through longer-term observation. In this study, the number of patients taking antihyperuricemic agents at baseline was small, so no clear differences in the results were observed in the analysis when antihyperuricemic agents were excluded; however, the presence of such treatment may introduce heterogeneity that may affect the trajectories of both uric acid and eGFR.

## Conclusion

This study revealed that in many cases, the administration of luseogliflozin to patients with type 2 diabetes resulted in a decrease in uric acid levels, irrespective of the degree of improvement in glycemic control.

In this study, the changes in uric acid levels were independent of changes in various metabolic parameters such as glycemic control and body weight. Δ uric acid was greater when the values prior to luseogliflozin administration were higher, and uric acid levels began to decrease after 1 month of treatment and were maintained for 12 months thereafter. In this study, changes in eGFR after luseogliflozin administration were significantly correlated with baseline eGFR, uACR, and changes in uric acid levels (Δ uric acid). The change in uric acid levels following SGLT2 inhibitor treatment was associated with metabolic parameters such as blood pressure and albuminuria, suggesting that it may function as a surrogate marker reflecting renal metabolic processes.
